# Metastable Polymers for Circular 3D Printing

**DOI:** 10.1002/adma.74682

**Published:** 2026-08-18

**Authors:** Johannes Markhart, Philipp Mainik, Eva Blasco

**Affiliations:** ^1^ Institute For Molecular Systems Engineering and Advanced Materials Heidelberg University Heidelberg Germany; ^2^ Institute of Organic Chemistry Heidelberg University Heidelberg Germany

**Keywords:** 3D printing, chemical circularity, digital light processing, metastable polymers, recycling, self‐immolative polymers

## Abstract

Reducing waste and enabling material reuse are central goals for sustainable advanced manufacturing. In light‐based 3D printing, this requires moving beyond permanent networks toward systems that can be easily returned to their original molecular building blocks, while preserving good performance. Here, a shift toward metastable, 3D printable polymers is introduced, which are intrinsically programmed for controlled depolymerization under mild conditions, enabling circular recovery. This concept is realized using self‐immolative polymers (SIPs), which undergo triggered, domino‐like depolymerization upon activation of a specific labile unit. These polymers are formulated for high‐resolution digital light processing 3D printing, yielding mechanically robust structures. Upon exposure to a defined trigger, the printed networks rapidly (in seconds) and completely disassemble under ambient conditions, regenerating their pristine monomers. These are recovered in near‐quantitative yield and subsequently reprocessed into chemically identical printable polymers. This metastability‐driven approach establishes a transformative pathway for circular 3D printing.

## Graphical abstract

3D‐printed polymers are usually permanent and hard to recycle. Here, metastable polymers are designed to fall apart on demand. A chemical trigger unzips the printed material back into its original building blocks within minutes at room temperature. These are recovered in pure form and reused to print again, enabling truly circular manufacturing.

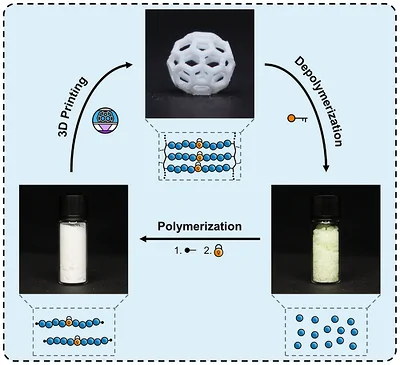

## 1 Introduction

Global plastic production consistently exceeds the capacity of existing recycling infrastructures, leading to a massive accumulation of waste in natural ecosystems [1]. The conventional linear economic model of “take‐make‐dispose” is inherently unsustainable because the qualities that make plastics valuable, namely durability and stability, simultaneously cause long‐lasting environmental pollution [2]. While mechanical recycling remains the most mature and energy‐efficient strategy, it is often unsuitable for contaminated waste streams or high‐performance materials due to significant property degradation upon reprocessing [3]. Consequently, the next grand challenge for polymer chemistry is the realization of chemical recycling to monomer, which allows for the recovery of pristine building blocks to enable true material circularity without loss of quality [4, 5, 6, 7, 8]. Establishing chemical recyclability is especially challenging for thermosetting polymers, as their permanent, 3D crosslinked networks are fundamentally incompatible with conventional recovery methods [9, 10, 11].

Additive manufacturing, also known as 3D printing, has emerged as a transformative strategy for processing these high‐performance thermoset materials [12]. The provided high degree of customizability and spatial freedom have led to substantial applications in specialized sectors such as microelectronics [13], personalized medicine [14], and soft robotics [15]. Among the diverse 3D printing techniques, vat photopolymerization approaches, such as digital light processing (DLP), represent the benchmark for high‐resolution manufacturing [16]. The versatility of this technique is being further expanded by recent developments in wavelength‐selective photocuring, which enable the integration of multiple materials within a single architecture [17, 18, 19]. In parallel, continuous liquid interface production [20, 21, 22] and emerging volumetric approaches [23, 24, 25] build upon these principles to achieve rapid and layerless 3D printing of complex geometries. Fundamental to all these strategies is the utilization of light‐curable liquid inks that undergo spatially controlled solidification to form solid 3D structures with exceptional precision and efficiency [26].

Despite the high degree of freedom in design, the mechanical robustness of printed parts typically relies on the irreversible crosslinking of multifunctional monomers into permanent networks. This creates a fundamental sustainability paradox, where the chemical bonds providing structural integrity simultaneously render the materials inherently infusible and unrecyclable, leading to the generation of persistent waste streams [27]. Initial sustainability efforts have introduced biobased feedstocks to reduce the carbon footprint of production [28, 29, 30]. However, this strategy generally results in the same irreversible network architectures, failing to provide a viable material recovery path at the end of life (Figure 1A).

**FIGURE 1:**
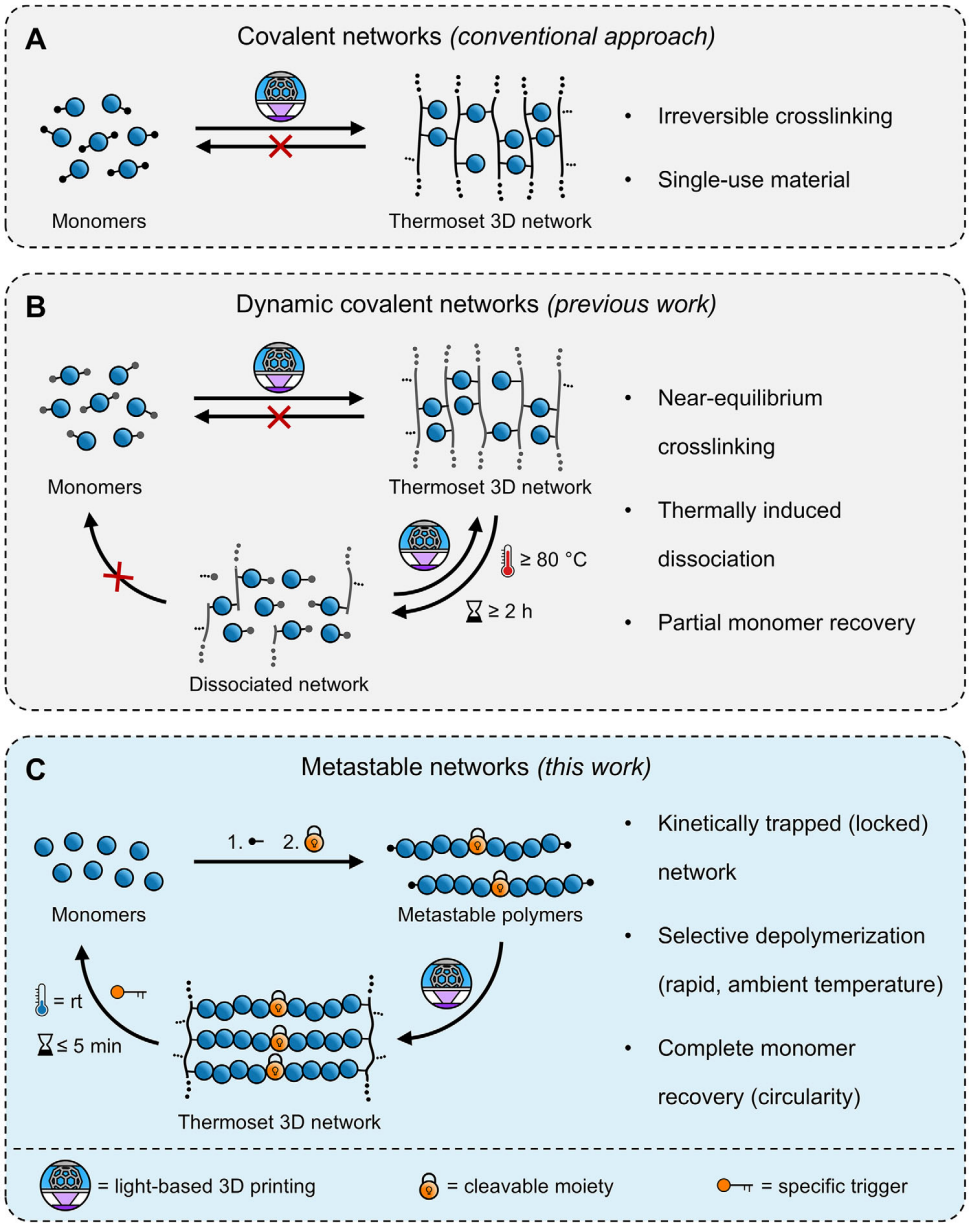
Material design strategies for light‐based 3D printing: toward truly circular systems. (A) Conventional materials form irreversible covalent bonds, yielding high structural integrity but enforcing a linear end‐of‐life material pathway. (B) Dynamic covalent networks based on dissociative exchange enable partial reversibility via thermally induced bond cleavage; however, their performance is constrained by thermal equilibrium limitations. (C) This work introduces printable metastable networks, in which the polymer backbones are kinetically trapped (locked) during use but can be selectively unlocked by a chemical trigger (key), initiating a rapid cascade depolymerization under ambient conditions to enable efficient monomer‐to‐monomer circularity.

A promising strategy to introduce recyclability into thermosets involves the integration of dynamic covalent bonds to enable network reprocessability [31, 32, 33, 34, 35, 36, 37]. In particular, recent advancements have led to the development of “circular” inks based on thermally accessible equilibria, in which the bonds formed during photopolymerization can be reversed to enable network dissociation. For example, Dove and coworkers leveraged the ring‐opening polymerization of lipoates to fabricate materials that are thermally depolymerizable upon phosphazene base catalysis [38]. In another approach, Xie and coworkers smartly utilized the acid‐mediated formation of dithioacetals to produce mechanically versatile networks capable of partial thermal dissociation into reprintable oligomers [39]. While these approaches represent significant progress, they remain fundamentally constrained by equilibrium‐limited depolymerization, resulting in dissociation products with substantial oligomeric content, thereby altering the performance of subsequent reprinting. Moreover, these processes demand extended reaction times of several hours at elevated temperatures (≥80°C), the addition of mediating reagents, and partially require manual pre‐processing, such as crushing or grinding the 3D objects, to accelerate degradation (Figure 1B) [38, 39].

In this work, we introduce a shift toward metastable printable polymers that are kinetically trapped during use yet thermodynamically predisposed to depolymerize into their constituent units at room temperature, enabling circular monomer recovery (Figure 1C). A particularly effective class of materials for this purpose is self‐immolative polymers (SIPs), which undergo a triggered, domino‐like depolymerization upon the cleavage of a specific end‐cap [40, 41, 42, 43, 44]. To this aim, we design SIPs featuring terminal photopolymerizable groups and a central cleavable moiety (lock), enabling the fabrication of high‐resolution architectures via DLP 3D printing that exhibit high thermal stability. Upon exposure to a specific trigger (key), the thermoset 3D network undergoes rapid (in seconds) and complete dissociation into its constituent monomers under ambient conditions. This fast unzipping process affords efficient recovery of pure monomers and allows resynthesis into prepolymers that are chemically identical to the pristine starting material. By decoupling operational stability from end‐of‐life disassembly through metastability, this work establishes a rapid and energy‐efficient pathway for the genuine chemical recycling of high‐performance 3D‐printed thermosets.

## 2 Results and Discussion

### 2.1 Design and 3D Printing of Metastable Polymers

To realize a metastable 3D printable platform, we selected the commercially available *o*‑phthalaldehyde (*o*PA) as the structural backbone to form a photoreactive prepolymer. Poly(*o*‑phthalaldehyde) (P*o*PA) is particularly interesting due to its low ceiling temperature (*T*_c_ ≈ –36°C), which provides the thermodynamic driving force for rapid and complete depolymerization back to the pristine monomer once the kinetic trap is removed [45, 46, 47]. In particular, we designed prepolymers that possess a cleavable unit and a photopolymerizable acrylate group at each terminus (P*o*PA‐dA) (Figure 2A), enabling their integration into a stable thermoset network upon 3D printing. The key to our design is the symmetrical coupling of two metastable chains around the central, cleavable moiety. For this purpose, a silyl ether was selected that can be selectively targeted by fluoride as a chemical trigger to enable efficient depolymerization [48, 49, 50].

**FIGURE 2:**
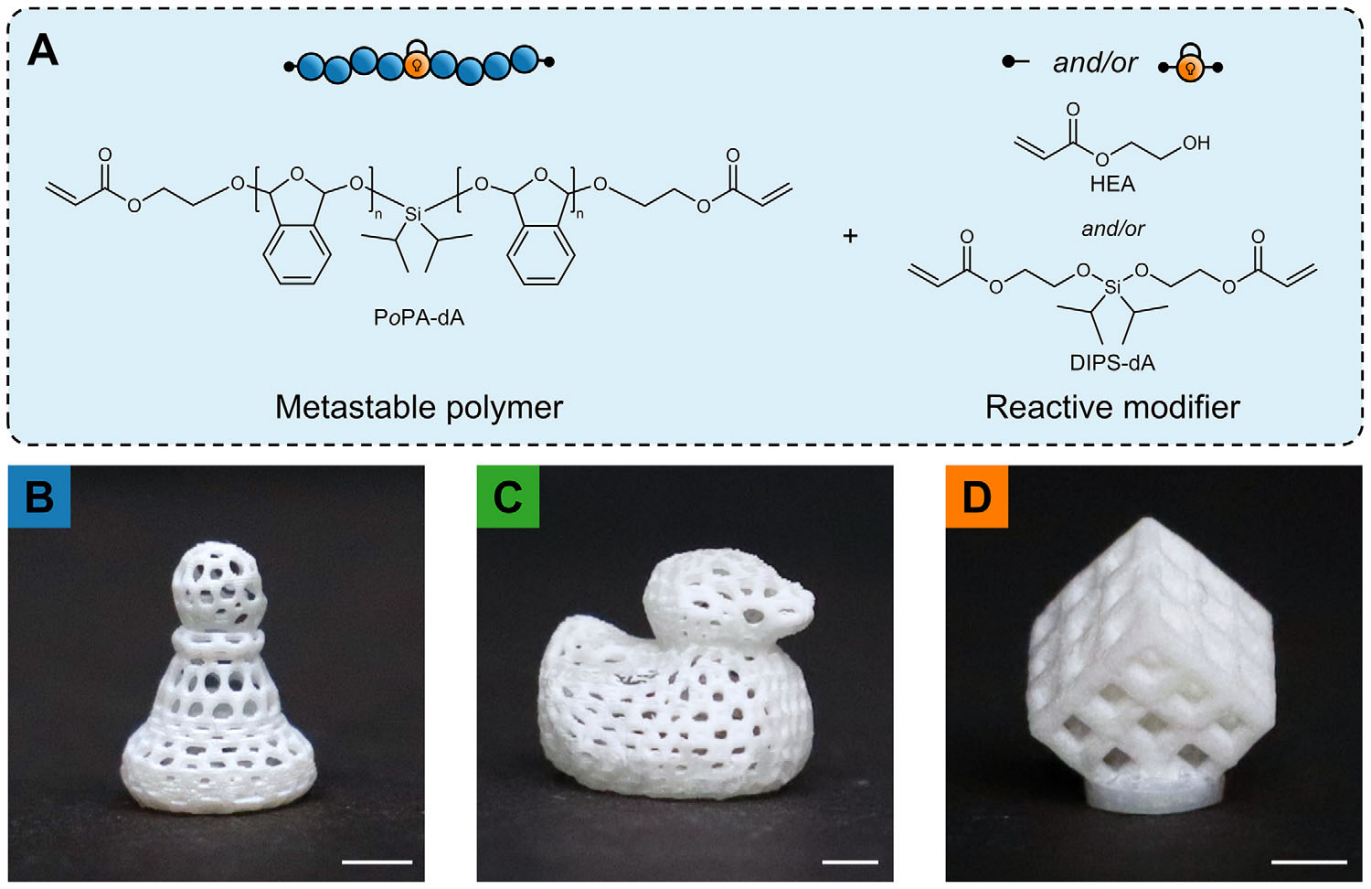
Metastable polymer platform for high‐resolution 3D printing. (A) Key components of the developed ink platform consisting of the photoreactive metastable prepolymer and an adjustable reactive modifier. The metastable polymer (P*o*PA‐dA) possesses a cleavable unit (orange) and a photopolymerizable acrylate group at each terminus, enabling the formation of a polymer network via 3D printing. Reactive modifiers (HEA and DIPS‐dA) are employed to tune the crosslinking and cleavable unit density of the final printed material. (B–D) Photographs of various centimeter‐scale 3D architectures featuring intricate geometries: a hollow chess figure (ink 1), a lattice‐surfaced duck (ink 2), and a complex cubic lattice (ink 3). These models demonstrate the platform's ability to resolve challenging features such as thin struts, internal voids, and high surface area‐to‐volume ratios with exceptional structural fidelity (scale bar = 2 mm).

Building on our previously established protocol [51], the prepolymers were prepared by anionic polymerization and obtained in high yields on a multi‐gram scale (Section S1.1). Importantly, the overall process sustainability was maintained through quantitative recovery of all solvents used in synthesis and purification (Figure S1). To provide the terminal photoreactive groups, chain growth was initiated from 2‐hydroxyethyl acrylate (HEA). Subsequent coupling of the metastable chains was achieved through the addition of dichlorodiisopropylsilane (DIPSCl_2_), enabling the bifunctional assembly around the central cleavable silyl unit. Size exclusion chromatography (SEC) confirmed successful coupling, revealing the expected molecular weight doubling compared to terminally silyl‐functionalized P*o*PA controls. The living character of the polymerization allowed for precise control over the target molecular weights and yielded prepolymers with narrow polydispersities (Figure S2). The sensitivity of the synthesized metastable prepolymers to the chemical trigger was validated via ^1^H‐NMR spectroscopy. When a fluoride salt was added to a P*o*PA‐dA solution at room temperature, it cleaved the silyl group, inducing a rapid, cascade‐like depolymerization to the *o*PA monomer within less than two minutes (Figure S3). This fast conversion from a macromolecule to its building blocks confirms the high reactivity and kinetic efficiency of the metastable design.

Next, the metastable prepolymer (P*o*PA‐dA) was exploited for DLP 3D printing, a vat photopolymerization technique favored for its exceptional resolution and rapid fabrication of complex architectures. Since the prepolymer is solid at room temperature, identifying a suitable solvent was critical. We selected dimethylisosorbide (DMI), a biobased, nontoxic, high‐boiling solvent that demonstrated good solvency for P*o*PA (up to 567 mg mL^–^^1^). This afforded homogeneous, transparent solutions suitable for light‐based 3D printing. To ensure efficient radical polymerization, we added the photoinitiator BAPO (1 wt%). Furthermore, we introduced small amounts of reactive modifiers (RMs) into the formulation (5 wt%) to tune crosslinking and cleavable unit density, and thus the performance of the printed material. In particular, we selected monofunctional HEA and a specifically difunctional, fluoride‐sensitive crosslinker (DIPS‐dA) (Figure 2A). The latter was synthesized by coupling two HEA units around a silyl functionality. The key advantage of this molecular design lies in the cleavable nature of all crosslinking sites within both the P*o*PA backbone and the DIPS‐dA crosslinker. On this basis, three distinct ink formulations were developed, containing P*o*PA‐dA as the main component, biobased DMI as a solvent, and differing by the employed RM: monofunctional HEA for ink 1, difunctional DIPS‐dA for ink 2, and a 50/50 wt% mixture for ink 3.

The curing kinetics were quantitatively assessed using Jacob's working curve model (Figure S4) [52], revealing a crosslinking density‐dependent decrease in critical curing energy. Accordingly, ink 2 (difunctional DIPS‐dA) exhibits the fastest curing, while ink 1 (monofunctional HEA) shows the slowest and ink 3 shows intermediate behavior. Importantly, all three developed inks demonstrated high photosensitivity, achieving rapid curing at a low light intensity of 1 mW cm^–^^2^. This highly energy‐efficient processing represents a significant advantage over previously reported dynamic covalent photopolymerizations that require substantially higher irradiation doses to ensure sufficient crosslinking.

The versatility of the metastable ink platform was demonstrated by the successful fabrication of various intricate, centimeter‐scale 3D architectures featuring challenging geometrical elements (Figure 2B–D). These models include a hollow chess figure, a duck with a complex lattice‐textured surface, and a dense cubic lattice, all of which resolve demanding features such as significant overhangs, thin struts, and internal voids with high structural fidelity. To further evaluate the precision of the system, we assessed the lateral resolution by printing line gratings with a width and spacing of only 27 µm. This corresponds to the pixel size of the employed DLP system and thus represents the technical resolution limit, confirming the exceptional spatial light control and minimal overcuring afforded by the metastable P*o*PA‐based inks (Figure S5).

### 2.2 Mechanical Tunability and Triggered Disassembly

Thermomechanical properties were investigated using dynamic mechanical analysis (DMA) in an oscillatory tensile configuration. We obtained storage moduli at room temperature ranging from 623 to 702 MPa, which scales directly with the crosslinking density adjusted via the reactive modifier ratio (Figure 3A, left), with the printed material using ink 2 containing the difunctional crosslinker yielding the highest stiffness. Despite the differences in storage moduli, all materials exhibited consistent glass transition temperatures between 74°C and 79°C (Figures S45, S47, and S49), reflecting the dominant influence of the P*o*PA backbone as the constant main component across all formulations. Mechanical performance was evaluated using tensile tests and revealed toughness values between 1.93 and 2.60 MJ m^–^^3^ (Figure 3A, middle). Consistent with the DMA results, we observed an increase in ultimate tensile strength (2.33–3.90 MPa) and a corresponding decrease in strain at break (114–89%) as the fraction of the bifunctional crosslinker (DIPS‐dA) increased (Figures S51–S53). A detailed comparison of these mechanical properties and the depolymerization characteristics with representative recyclable 3D printable systems is provided in Table S1 (Section S3).

**FIGURE 3:**
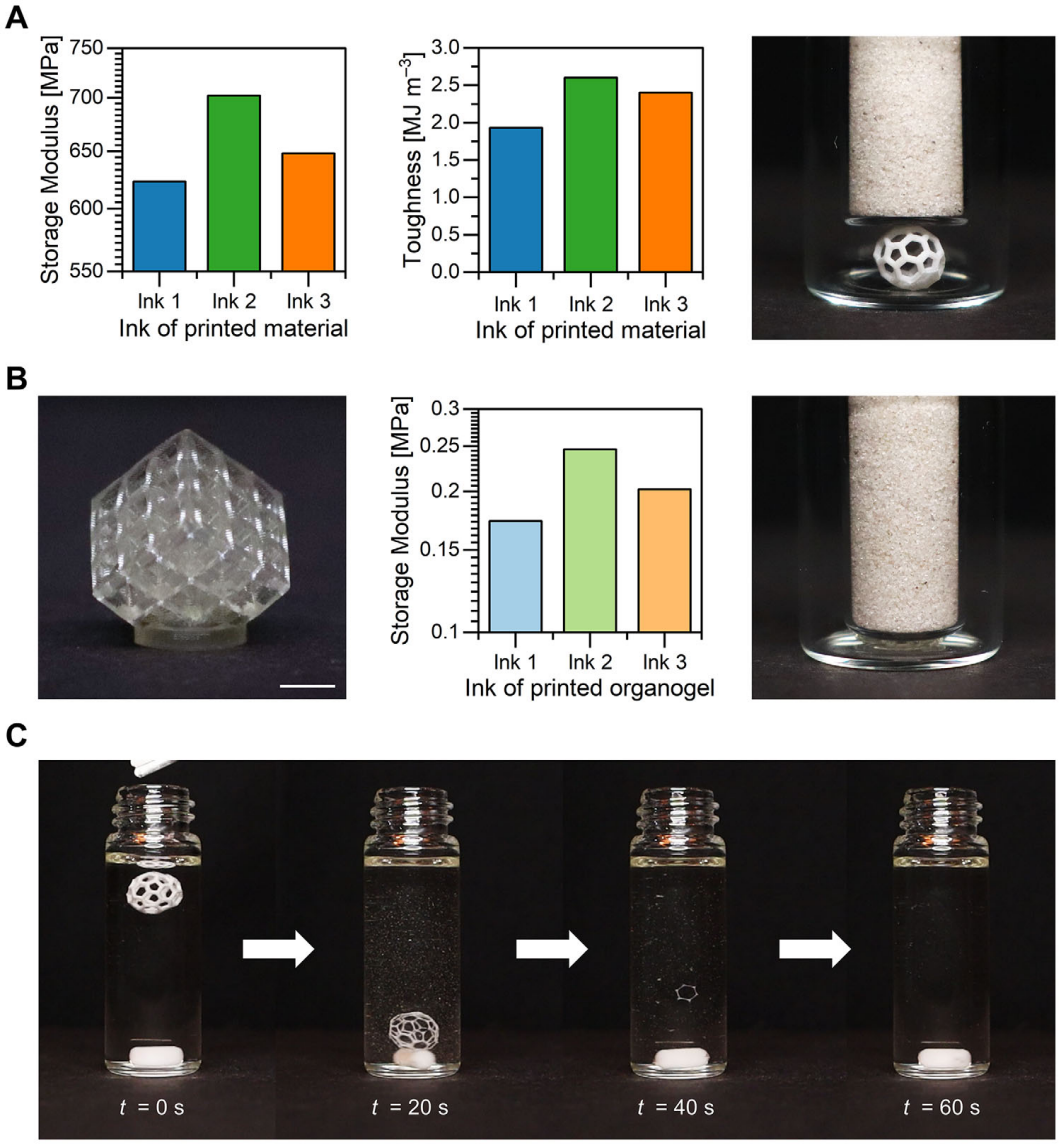
Mechanical properties and on‐demand deconstruction of the metastable platform. (A) Mechanical evaluation of the printed ink library. Storage moduli at 25°C (left) and tensile toughness values (middle) demonstrate the crosslinking density‐dependent modulation of mechanical properties through reactive modifier variation. A load‐bearing test (right) on a printed buckyball (ink 1) shows no perceptible deformation under a static load of 50 mN. (B) Characterization of the organogel state after development in DMI. Photograph of a 3D printed lattice cube (ink 2) in the transparent organogel state (left, scale bar = 2 mm). Strong solvophilic interactions trap residual solvent within the network, leading to a pronounced plasticizing effect that affords soft and elastic properties, as evidenced by the corresponding storage moduli at 25°C (middle). Load‐bearing (right) under identical conditions on an organogel buckyball (ink 1) results in significant elastic compression (see details in Section S4). (C) Time‐lapse photography demonstrating the rapid depolymerization of a 3D‐printed buckyball (ink 2) in a 1 m TBAF/THF solution at room temperature, achieving complete network dissociation within 60 s.

To further tune the mechanical properties, the as‐printed 3D objects were developed in the same biobased solvent included in the formulation (DMI) to remove unreacted species, followed by drying under vacuum (Figure S6). A significant fraction of the solvent remained retained within the structure, enabling the fabrication of soft, elastic functional materials (Figure 3B, left). This behavior arises from the pronounced plasticizing effect of the retained DMI, resulting in a material with storage moduli in the kPa range (173–246 kPa) (Figure 3B, middle). The resulting mechanical disparity in comparison to the 3D printed materials without remaining DMI was demonstrated by load‐bearing tests (Figure 3A,B, right). These results demonstrate that our metastable platform offers a pronounced mechanical versatility.

Having established the 3D printable platform, we systematically evaluated its depolymerization behavior, leveraging the inherent metastability of the P*o*PA‐based network to enable monomer recovery and therefore, circularity. To this aim, 3D‐printed structures were immersed in a solution containing the trigger (fluoride salt). Rapid and complete deconstruction was observed at ambient temperature within minutes (Figure 3C). Notably, we observed a formulation‐dependent kinetic profile, with materials derived from ink 2 disassembling the fastest (<1 min), while ink 1 exhibited the relatively “slowest” response (<5 min) (Movie S1). This correlation is attributed to the varying density of fluoride‐sensitive silyl sites within the network. As the DIPS‐dA content increases, the number of triggerable points for cascade initiation rises, thereby accelerating the unzipping of the entire architecture. To verify the high selectivity of fluoride as the depolymerization trigger, the 3D printed architectures were exposed to various harsh environments, including strong acidic, basic, and saline aqueous solutions. The structures remained intact under all these conditions (see details in Section S5). Furthermore, thermogravimetric analysis (TGA) was employed to assess the thermal stability of the printed materials, revealing a degradation onset temperature of about 200°C. These results demonstrate that the material remains intact under harsh conditions and thermal stress, depolymerizing only in response to its specific trigger.

### 2.3 Circular Monomer Recovery and Reuse

To demonstrate true circularity, we evaluated the *o*PA monomer recovery and its resynthesis into photoreactive prepolymers for reuse (Figure 4A). In detail, three distinct strategies were employed for isolation of repolymerizable *o*PA. First, chromatography on silica was performed, utilizing polarity differences to isolate pristine *o*PA in 94% yield, which subsequently afforded the P*o*PA prepolymer in 90% yield upon repolymerization. Secondly, the depolymerization crude was plugged over silica and suspended in petroleum ether, followed by filtration to yield pure monomer (90%) and chemically identical prepolymer (89%). Third, sublimation of the monomer allowed for clean recovery of *o*PA (85%) and P*o*PA resynthesis (92%) as well (Figure 4B). The recovered monomer amounts are in close agreement with the theoretically expected *o*PA content of the printed objects, assuming equivalent incorporation of all photoreactive ink components (see details in Section S6). The fidelity of this circular process was validated by comparing the resynthesized prepolymers to their pristine counterparts. SEC analysis confirmed that the molecular weight distribution remained remarkably consistent, with nearly identical retention times observed across all strategies (Figure 4C). Furthermore, ^1^H‐NMR spectroscopy confirmed the identical chemical composition of the recycled material, exhibiting the characteristic aromatic and acetal backbone signals and maintaining a similar acrylate‐to‐backbone ratio (Figure 4D).

**FIGURE 4:**
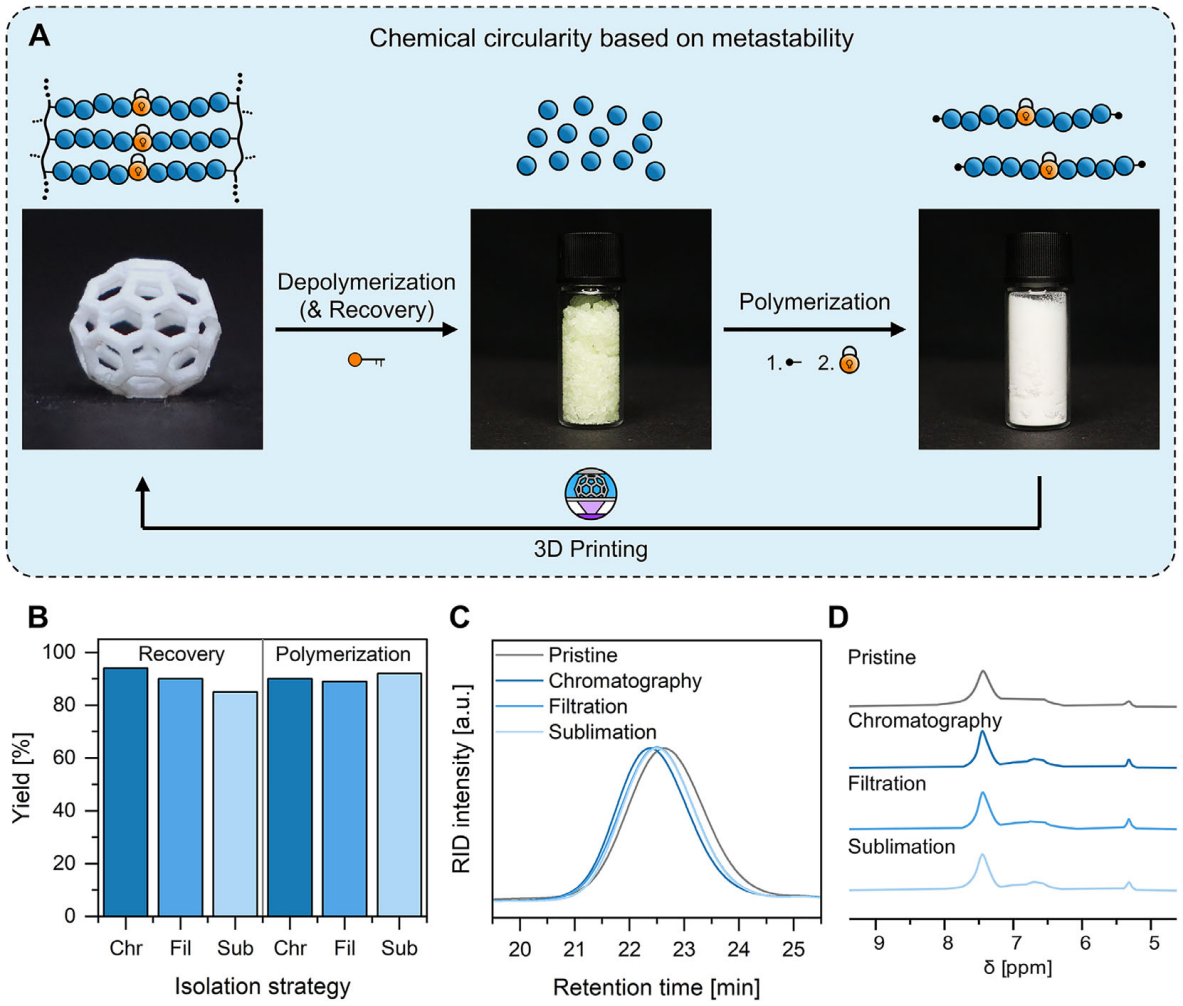
Metastable platform enabling full chemical circularity. (A) A 3D‐printed structure undergoes selective‐triggered depolymerization, yielding the starting crystalline monomer *o*PA. The recovered monomer is subsequently resynthesized into the original P*o*PA‐dA prepolymer, thereby closing the material loop for reprinting. (B) Yields for monomer recovery and prepolymer resynthesis utilizing three distinct isolation strategies: chromatography (Chr), filtration (Fil), and sublimation (Sub). (C) SEC chromatograms comparing pristine and resynthesized P*o*PA, confirming identical molecular weight distributions and elution behavior across all recycling routes. (D) ^1^H‐NMR spectra (DCM‐d_2_) of pristine and resynthesized P*o*PA prepolymers, demonstrating the preservation of molecular identity.

Importantly, the completeness of the depolymerization process afforded by the inherent metastability of P*o*PA circumvents limitations of previous systems based on oligomeric residuals, which led to altered curing kinetics or degraded mechanical properties. In our system, the reformed networks are genuinely identical to the pristine materials at the molecular level. Collectively, these results establish a high‐yield, “monomer‐to‐prepolymer” loop that preserves material quality and molecular identity, successfully overcoming the degradation of properties often seen in iterative recycling of 3D printed thermosets. To confirm that this molecular fidelity translates into preserved processing performance, an ink was reformulated from the recycled prepolymers and reprinted. The resulting material reproduced the curing kinetics, lateral resolution, and triggered deconstruction of the pristine system, thereby closing the loop not only at the molecular but also at the functional level (see details in Section S7).

## 3 Conclusion

In summary, we have established a transformative strategy for chemical circularity in 3D printing by utilizing metastable SIPs. We successfully synthesized photoreactive P*o*PA prepolymers featuring a fluoride‐sensitive silyl core and formulated them into inks for high‐performance DLP 3D printing. Our strategy effectively decouples operational stability from end‐of‐life deconstruction: while the P*o*PA backbone is kinetically trapped in a functional state during use, it remains thermodynamically programmed for a rapid, cascade‐like breakdown upon activation. By modulating the crosslinking density via specifically tailored reactive modifiers, we achieved mechanical properties and toughness values that set a new benchmark among recyclable thermosets for 3D printing.

The developed ink platform enables the fabrication of complex, high‐resolution 3D architectures with exceptional structural fidelity at low exposure energies. The most compelling advantage of our approach is the unparalleled depolymerization kinetics: upon chemical triggering, the 3D objects undergo complete network dissociation within only 1 to 5 min at ambient temperature. This represents a substantial advancement compared to existing circular 3D printing strategies that are constrained by equilibrium‐limited dissociation, elevated temperatures, and prolonged reaction times. Furthermore, through diverse and scalable isolation routes, we demonstrated the nearly quantitative recovery of pristine *o*PA monomers, facilitating high‐yield resynthesis into chemically identical prepolymers and thus closing the material loop without degradation of quality. The availability of these diverse isolation strategies underscores the robustness and practical versatility of our platform, as the recycling method can be selected based on available laboratory infrastructure or specific requirements. Importantly, an ink reformulated from the recycled prepolymers reproduced the curing behavior, printing resolution, and triggered deconstruction of the pristine material, confirming that the recovered building blocks are fully reprintable and that circularity is preserved at both the molecular and the functional level.

Looking ahead, we are confident that the implementation of metastability will serve as a versatile design template for the next generation of chemically circular materials. While the potential of metastable polymers has been recognized for years, their practical application has often been restricted by processing challenges. Beyond the poly(*o*‐phthalaldehyde) backbone demonstrated here, alternative metastable polymers and composite formulations could be employed to tune mechanical properties independently of the depolymerization mechanism, while retaining the underlying lock and key concept. Given its rapid, mild, and complete depolymerization, this platform could enable more sustainable applications in advanced manufacturing by facilitating fast, efficient, and complete recovery of valuable polymer components. Such capabilities may be particularly beneficial for applications in soft robotics, electronics, and other rapidly evolving technologies where end‐of‐life recovery and material circularity are increasingly important. We envision that the expansion of this strategy toward new metastable backbones and triggering systems will unlock new frontiers in sustainable manufacturing and effectively bridge the gap between fundamental polymer research and real‐world applicability.

## 4 Experimental Section

### 4.1 Materials

2‐Hydroxyethyl acrylate (HEA, 96%, containing 200–650 ppm MEHQ as inhibitor), anhydrous pyridine (99.8%), tetrabutylammonium fluoride (TBAF) solution (1 m in THF), dimethyl isosorbide (DMI, ≥ 99%), phenylbis(2,4,6‐trimethylbenzoyl)phosphine oxide (BAPO, 97%), 2,6‐di‐*tert*‐butyl‐4‐methylphenol (BHT, ≥ 99.0%), and triethylamine (≥ 99.5%) were purchased from *Merck*. 1,8‐Diazabicyclo(5.4.0)undec‐7‐ene (DBU, 98+%), tetrahydrofuran (THF, extra dry over molecular sieve, 99.5%, containing 200 ppm BHT as stabilizer), and benzyl mercaptan (BnSH, 99%) were obtained from *Fisher Scientific*. Dichlorodiisopropylsilane (DIPSCl_2_, > 98.0%) was acquired from *TCI*. The used *o*‐phthalaldehyde (*o*PA, 99.75%) was purchased from *BLDpharm*. Tetrahydrofurane‐d_8_ (THF‐d_8_, 99.5%), dichloromethane‐d_2_ (DCM‐d_2_, 99.6%), and chloroform‐d (99.8%) were obtained from *Deutero*. All reagents were used without further purification. Unless otherwise stated, all reactions were performed in a yellow‐light environment and under an inert nitrogen atmosphere by using common Schlenk techniques and flame dried glassware.

### 4.2 Size Exclusion Chromatography (SEC)

SEC measurements were performed on a *Shimadzu Nexera LC‐40* system (with LC‐40D pump, autosampler SIL‐40C, DGU‐403 (degasser), CBM‐40 (controlling unit), column oven CTO‐40C, UV‐detector SPD40, and RI‐detector RID‐20A). The system was equipped with a PSS GRAM column set consisting of a guard column (8 × 50 mm) and two analytical linear columns (8 × 300 mm) with a particle size of 10 µm. The measurements were performed in DMAc with LiBr (1 g L^−1^) at a flow speed of 1 mL min^−1^ and a temperature of 50°C. Calibration was performed against different poly(methyl methacrylate) standards (800–988 000 Da, PMMA). The obtained chromatograms were examined using the *Shimadzu LabSolutions* software. To prevent polymerization of acrylated P*o*PA during SEC measurements, an addition with benzyl mercaptan was conducted beforehand. This procedure is described in detail in the Supporting Information.

### 4.3 Nuclear Magnetic Resonance (NMR) Spectroscopy

To confirm the purity and identity of synthesized compounds, NMR spectroscopy was employed. ^1^H‐NMR spectra were recorded with a *Bruker Avance III 300*, *Βruker Avance III 400*, *Bruker Avance III 600*, or *Nanalysis‐100* NMR spectrometer and referenced to the solvent in use. ^13^C‐ and ^19^F‐NMR spectra were recorded with a *Bruker Avance III 400* and *Bruker Avance III 300* NMR spectrometer, respectively. Small‐molecule and polymeric samples were measured with relaxation delays of 0.1 s and 1 s, respectively. To analyze and process the data, the *MestreLab Research MestReNova 16.0.0* software was employed. Chemical shifts are reported as dimensionless δ values in ppm and coupling constants *J* are given in Hertz (Hz). The apparent resonance multiplicity was reported as s (singlet), br s (broad singlet), dd (doublet of doublets), or m (multiplet).

### 4.4 Ink Formulation

While comprehensive preparation procedures for the ink library are provided in the Supporting Information, the preparation of ink 1 is described here as a representative protocol. First, P*o*PA‐dA (1.24 g, 31.0 wt%, 1.1 mol%) was dissolved in DMI (2.19 mL, 2.52 g, 62.9 wt%, 87.9 mol%). Next, HEA (181 µL, 200 mg, 5.0 wt%, 10.5 mol%), BAPO (40 mg, 1.0 wt%, 0.6 mol%), and BHT (4 mg, 0.1 wt%, 0.1 mol%) were added. To ensure a complete solution, the ink was sonicated at rt for 30 min.

### 4.5 Digital Light Processing (DLP)

DLP 3D printing was conducted using an *Asiga MAX X27* UV printer with an LED light source of 385 nm and a pixel resolution of 27 µm. To evaluate the printing parameters of each ink, Jacobs' protocol was followed [52]. In addition to the determined necessary irradiation time of the respective ink to cure the chosen z‐layer thickness of 100 µm, the objects were printed with an offset of 180 µm to ensure sufficient interlayer bonding. To print 3D objects, a custom aluminum build platform with a surface area of 25 mm × 25 mm was used along with a custom aluminum vat. Printing was performed at rt in a yellow‐light environment. The fabricated structures were suspended in DMI at rt for 2 min and postcured utilizing an *Asiga Flash* UV chamber for at rt for 10 min. Subsequently, the structures were either directly dried in vacuo to afford the organogel state or sonicated in THF and EtOH at rt and dried in vacuo to afford the solvent‐free state. To slice the STL files of all printed structures with the specified z‐layer thickness, the *Asiga Composer* software was used.

### 4.6 Optical Microscopy

High‐resolution images of the DLP 3D‐printed rectangular block with engraved line gratings to assess the lateral resolution were obtained with a *Zeiss Axio Imager M2* microscope equipped with an *EC Plan‐Neofluar 10×/0.3 Korr Ph* objective and an *Axiocam 705* microscope camera. All images were recorded in transmission mode. The images were processed with the *Zeiss Zen* software.

### 4.7 Mechanical and Thermomechanical Characterization

Comprehensive mechanical characterization was performed using a *TA Instruments DMA 850* in a tensile configuration. For dynamic mechanical analysis (DMA), 3D printed strips (20 mm × 5 mm × 1 mm) were analyzed at a frequency of 1 Hz and a heating rate of 5 K min^–^^1^. To ensure measurements were conducted within the linear viscoelastic region, the oscillation amplitude was tailored to the material state: 1% for the soft organogel specimens (scanned from 20 to 35°C) and 0.05% for the stiff dry samples (scanned from 0 to 150°C). Uniaxial tensile properties were evaluated using 3D printed dumbbell‐shaped specimens (external dimensions: 20 mm × 6 mm × 1 mm). Measurements were performed until complete mechanical failure to determine the ultimate tensile strength (*σ*_UTS_), elongation at break (*ε*_b_), and toughness (*U*_T_). To account for the significantly different mechanical profiles, strain ramps were specifically adjusted: a rate of 5% min^–^^1^ was employed for the elastic organogels, while a slower rate of 2% min^–^^1^ was utilized for the dry materials.

### 4.8 Thermogravimetric Analysis (TGA)

TGA was conducted with a *Mettler Toledo TGA 2* instrument in the range of 30 to 600°C with a heating rate of 10 K min^− 1^ and under an inert atmosphere.

### 4.9 Triggered Depolymerization

For the qualitative demonstration of rapid deconstruction (Figure 3C, Movies S1–S2), 3D printed buckyballs were suspended in a 1 m solution of TBAF in THF (4 mL) and stirred at rt for 1–5 min to ensure complete network dissociation. For the quantitative assessment of monomer recovery, the protocol was adapted for resource efficiency using a lower trigger concentration and extended reaction time. To demonstrate the universality of the recycling process, representative mixtures of samples printed with all three inks (250 mg total) were suspended in THF (4.3 mL). A TBAF solution (1 m in THF, 480 µL) was added and the mixture was stirred at rt for 15 min. Yields for the subsequent purification steps were calculated based on the theoretical *o*PA content of the printed architectures, assuming equal photopolymerization speed of all acrylated ink components.

### 4.10 Chromatography

The depolymerization crude was concentrated in vacuo and purified using column chromatography (SiO_2_, *n*‐hexane/ethyl acetate 3:1). After removal of the eluent and drying in vacuo, pure *o*PA (192 mg, 77 wt% relative to total object mass, 94% recovery yield) was isolated as a colorless, crystalline solid.

### 4.11 Filtration

The depolymerization crude was poured on a silica plug (5 cm) and eluted with THF (100 mL) to remove polar side products and residual salts. After removal of the eluent and drying in vacuo, the residue was suspended in cooled petroleum ether (5 mL). The resulting suspension was filtered, washed with cooled petroleum ether (3 × 2 mL), and dried in vacuo to afford pure *o*PA (185 mg, 74 wt% relative to total object mass, 90% recovery yield) as a colorless, crystalline solid.

### 4.12 Sublimation

The depolymerization crude was concentrated in vacuo and the *o*PA monomer was isolated using vacuum sublimation (60°C, 0.4 mbar). This afforded pure *o*PA (175 mg, 70 wt% relative to total object mass, 85% recovery yield) as a colorless, crystalline solid.

### 4.13 Resynthesis of Recycled Prepolymers

The isolated *o*PA monomers from all three routes were utilized for the resynthesis of photoreactive P*o*PA‐dA following the representative polymerization protocol at a scale of 150 mg of monomer.

## Author Contributions

**Johannes Markhart**: conceptualization, investigation, methodology, writing – original draft, writing – review and editing, validation, visualization, formal analysis, data curation. **Philipp Mainik**: conceptualization, investigation, methodology, writing – review and editing. **Eva Blasco**: conceptualization, investigation, funding acquisition, writing – original draft, methodology, writing – review and editing, project administration, supervision, resources, visualization.

## Funding

Deutsche Forschungsgemeinschaft (DFG, German Research Foundation) via the Excellence Cluster “3D Matter Made to Order” (EXC‐2082/2‐390761711), Carl Zeiss Foundation through the “Carl‐Zeiss Foundation‐Focus@HEiKA”, Fonds der Chemischen Industrie through a Kekulé Fellowship, and the data storage service “SDS@hd” supported by the Ministry of Science, Research and the Arts Baden‐Württemberg (MWK) and the DFG (INST 35/1503‐1 FUGG).

## Conflicts of Interest

The authors declare no conflicts of interest.

## Supporting Information

Supporting Information is available from the Wiley Online Library or from the author.

## Supporting information




**Supporting File 1**: adma74682‐sup‐0001‐SuppMat.docx.

**Supporting File 2**: adma74682‐sup‐0002‐MovieS1.mp4.

**Supporting File 3**:adma74682‐sup‐0003‐MovieS2.mp4

## Data Availability

The data that support the findings of this study are openly available in heiDATA, a data repository of Heidelberg University at https://doi.org/10.11588/DATA/LLDGPE.
