# Supplementary material for: Metastable Polymers for Circular 3D Printing
Source: Adv Mater. 2026 Aug 18;38(58):e74682. doi: 10.1002/adma.74682 (PMC13674621; doi:10.1002/adma.74682)
Supplement: Supplementary file 1 [file ADMA-38-e74682-s002.docx]

Supporting Information

Metastable Polymers for Circular 3D Printing

Johannes Markhart, Philipp Mainik, and Eva Blasco*

**Table of Contents**

S1. Synthesis and characterization of metastable prepolymers	2

S1.1. Synthetic protocols	2

S1.2. Solvent recovery, prepolymer architecture, and depolymerization	8

S2. Ink formulation and 3D printing performance	11

S2.1. Ink formulations	11

S2.2. DLP 3D printability and lateral resolution assessment	12

S3. Comparison with recyclable 3D printable materials	14

S4. Organogel development and mechanical profile	16

S5. Depolymerization trigger selectivity	18

S6. Network breakdown mechanism and recovery efficiency	19

S7. Reprinting and property retention of recycled prepolymers	21

S8. Appendix	24

S9. References	45

# S1. Synthesis and characterization of metastable prepolymers

## S1.1. Synthetic protocols

**Poly-*o*-phthalaldehyde (P*o*PA-dA), average *M*_n_ = 4369 g mol^–1^, *DP*_n_ = 30**

**Scheme S1:** Synthesis of P*o*PA-dA (*DP*_n_ = 30).

Following a modified version of our previously reported procedure,^[1]^ *o*-phthalaldehyde (3.19 g, 23.8 mmol, 25.0 equiv.) and 2-hydroxyethyl acrylate (200 µL, 221 mg, 1.90 mmol, 2.00 equiv.) were suspended in dry THF (40 mL) and the resulting mixture was cooled to
–84 °C using liquid N_2_ and ethyl acetate. 1,8-Diazabicyclo(5.4.0)undec-7-ene (1.42 mL, 1.45 g, 9.52 mmol, 10.00 equiv.) was added and the reaction mixture was stirred at –84 °C for 8 h. Next, anhydrous pyridine (3.84 mL, 3.77 g, 47.6 mmol, 50.0 equiv.) and dichlorodiisopropylsilane (1.42 mL, 1.45 g, 9.52 mmol, 5.00 equiv.) were added. Under subsequent stirring for 14 h, the mixture was allowed to warm to room temperature. Afterwards, the reaction mixture was added to cooled EtOH (400 mL) and the resulting suspension was filtered. The residue was washed with cold EtOH (3 × 50 mL) and dried *in vacuo* to afford P*o*PA-dA (2.72 g, 624 µmol, 77%) as a colorless, crystalline solid.

**^1^H-NMR** (100 MHz, DCM-d_2_, 306 K): δ = 7.97–7.43 (m, *o*PA-CH_Ar_), 6.98–6.05 (m, *o*PA-CHO and 2H HEA-CH=CH_2_), 5.80 (br s, 1H, HEA-CH=CH_2_), 4.35 (br s, 2H, HEA-CH_2_), 3.86 (br s, 2H, HEA-CH_2_), 1.20 (br s, DIPS-CH and -CH_3_).

**^1^H-NMR** (301 MHz, CDCl_3_, 299 K) after depolymerization: δ = 10.55 (s, 60H, *o*PA-CHO), 8.07–7.91 (m, 60H, *o*PA-CH_Ar_), 7.86–7.71 (m, 60H, *o*PA-CH_Ar_), 6.46 (dd, *J* = 17.3, 1.4 Hz, 2H, HEA-CH=CH_2_), 6.17 (dd, *J* = 17.3, 10.3 Hz, 2H, HEA-CH=CH_2_), 5.87 (dd, *J* = 10.3, 1.5 Hz, 2H, HEA-CH=CH_2_), 4.36–4.25 (m, 4H, HEA-CH_2_), 3.93–3.81 (m, 4H, HEA-CH_2_), 1.13–0.92 (m, 14H, DIPS-CH and -CH_3_).

**Poly-*o*-phthalaldehyde (P*o*PA-dA), average *M*_n_ = 7051 g mol^–1^, *DP*_n_ = 50**

**Scheme S2:** Synthesis of P*o*PA-dA (*DP*_n_ = 50).

Following a modified version of our previously reported procedure,^[1]^ *o*-phthalaldehyde (3.19 g, 23.8 mmol, 50.0 equiv.) and 2-hydroxyethyl acrylate (100 µL, 111 mg, 953 µmol, 1.00 equiv.) were suspended in dry THF (40 mL) and the resulting mixture was cooled to −84 °C using liquid N_2_ and ethyl acetate. 1,8-Diazabicyclo(5.4.0)undec-7-ene (712 µL, 725 mg, 4.76 mmol, 10.00 equiv.) was added and the reaction mixture was stirred at −84 °C for 8 h. Next, anhydrous pyridine (1.92 mL, 1.88 g, 23.8 mmol, 50.0 equiv.) and dichlorodiisopropylsilane (430 µL, 441 mg, 2.38 mmol, 5.00 equiv.) were added. Under subsequent stirring for 15 h, the mixture was allowed to warm to room temperature. Afterwards, THF was removed under reduced pressure and the residue was suspended in EtOH (200 mL). The resulting suspension was filtered, washed with EtOH (3 × 50 mL), and dried *in vacuo* to afford P*o*PA (3.32 g, 471 µmol, 99%) as a colorless, crystalline solid.

**^1^H-NMR** (100 MHz, DCM-d_2_, 306 K): δ = 7.91–7.13 (m, *o*PA-CH_Ar_), 7.05–6.08 (m, *o*PA-CHO and 2H HEA-CH=CH_2_), 5.88 (br s, 1H, HEA-CH=CH_2_), 4.35 (br s, 2H, HEA-CH_2_), 3.84 (br s, 2H, HEA-CH_2_), 1.17 (br s, DIPS-CH and -CH_3_).

**^1^H-NMR** (301 MHz, CDCl_3_, 298 K) after depolymerization: δ = 10.54 (s, 100H, *o*PA-CHO), 8.06–7.89 (m, 100H, *o*PA-CH_Ar_), 7.88–7.68 (m, 100H, *o*PA-CH_Ar_), 6.45 (dd, *J* = 17.3, 1.5 Hz, 2H, HEA-CH=CH_2_), 6.16 (dd, *J* = 17.3, 10.4 Hz, 2H, HEA-CH=CH_2_), 5.87 (dd, *J* = 10.4, 1.6 Hz, 2H, HEA-CH=CH_2_), 4.34–4.26 (m, 4H, HEA-CH_2_), 3.93–3.84 (m, 4H, HEA-CH_2_), 1.11–1.00 (m, 14H, DIPS-CH and -CH_3_).

**Poly-*o*-phthalaldehyde (P*o*PA-dA), average *M*_n_ = 15904 g mol^–1^, *DP*_n_ = 116**

**Scheme S3:** Synthesis of P*o*PA-dA (*DP*_n_ = 116).

Following a modified version of our previously reported procedure,^[1]^ *o*-phthalaldehyde (3.19 g, 23.8 mmol, 50.0 equiv.) and 2-hydroxyethyl acrylate (50.0 µL, 55.3 mg, 476 µmol, 2.00 equiv.) were suspended in dry THF (40 mL) and the resulting mixture was cooled to
–84 °C using liquid N_2_ and ethyl acetate. 1,8-Diazabicyclo(5.4.0)undec-7-ene (356 µL, 363 mg, 2.38 mmol, 10.00 equiv.) was added and the reaction mixture was stirred at –84 °C for 8 h. Next, anhydrous pyridine (959 µL, 942 mg, 11.9 mmol, 50.0 equiv.) and dichlorodiisopropylsilane (215 µL, 220 mg, 1.19 mmol, 5.00 equiv.) were added. Under subsequent stirring for 15 h, the mixture was allowed to warm to room temperature. Afterwards, the reaction mixture was added to cooled EtOH (400 mL) and the resulting suspension was filtered. The residue was washed with cold EtOH (3 × 50 mL) and dried *in vacuo* to afford P*o*PA-dA (3.12 g, 196 µmol, 95%) as a colorless, crystalline solid.

**^1^H-NMR** (100 MHz, DCM-d_2_, 306 K): δ = 7.92–7.13 (m, *o*PA-CH_Ar_), 7.09–6.11 (m, *o*PA-CHO and 2H HEA-CH=CH_2_), 5.87 (br s, 1H, HEA-CH=CH_2_), 4.34 (br s, 2H, HEA-CH_2_), 3.83 (br s, 2H, HEA-CH_2_), 1.14 (br s, DIPS-CH and -CH_3_).

**^1^H-NMR** (301 MHz, CDCl_3_, 299 K) after depolymerization: δ = 10.55 (s, 232H, *o*PA-CHO), 8.03–7.92 (m, 232H, *o*PA-CH_Ar_), 7.84–7.74 (m, 232H, *o*PA-CH_Ar_), 6.46 (dd, *J* = 17.3, 1.5 Hz, 2H, HEA-CH=CH_2_), 6.17 (dd, *J* = 17.2, 10.3 Hz, 2H, HEA-CH=CH_2_), 5.87 (dd, *J* = 10.4, 1.6 Hz, 2H, HEA-CH=CH_2_), 4.33–4.29 (m, 4H, HEA-CH_2_), 3.90–3.86 (m, 4H, HEA-CH_2_), 1.07–1.01 (m, 14H, DIPS-CH and -CH_3_).

**Poly-*o*-phthalaldehyde mercaptan-adduct (P*o*PA-dBnS), average *M*_n_ = 7300 g mol^–1^, *DP*_n_ = 50**

**Scheme S4:** Synthesis of P*o*PA-dBnS (*DP*_n_ = 50).

Following a modified version of our previously reported procedure,^[1]^ poly-*o*-phthalaldehyde (average *M*_n_ = 7051 g mol^– 1^, 250 mg, 35.5 µmol, 1.00 equiv.) and benzyl mercaptan (41.6 µL, 44.0 mg, 355 µmol, 10.00 equiv.) were suspended in dry THF (1 mL). Diazabicyclo(5.4.0)undec-7-ene (5.30 µL, 5.40 mg, 35.5 µmol, 1.00 equiv.) was added and the resulting mixture was stirred at rt for 28 h. Subsequently, the reaction mixture was added to EtOH (20 mL) and the resulting suspension was filtrated. The residue was washed with EtOH (3 × 5 mL) and dried *in vacuo* to afford P*o*PA-dBnS (164 mg, 22.5 µmol, 63%) as a colorless, crystalline solid.

**^1^H-NMR** (100 MHz, DCM-d_2_, 306 K): δ = 7.92–7.13 (m, *o*PA-CH_Ar_ and BnS-CH_Ar_),
6.98–6.32 (m, *o*PA-CHO), 4.27 (br s, 4H, HEA-CH_2_), 3.88 (br s, 4H, HEA-CH_2_), 3.72 (br s, 4H, BnS-CH_2_), 2.64 (br s, 8H, HEA-CH_2_), 1.15 (br s, DIPS-CH and -CH_3_).

**^1^H-NMR** (301 MHz, CDCl_3_, 298 K) after depolymerization: δ = 10.54 (s, 100H, *o*PA-CHO), 8.03–7.94 (m, 100H, *o*PA-CH_Ar_), 7.83–7.74 (m, 100H, *o*PA-CH_Ar_), 7.35–7.28 (m, 10H, BnS-CH_Ar_), 4.28–4.21 (m, 4H, HEA-CH_2_), 3.84–3.79 (m, 4H, HEA-CH_2_), 3.74 (br s, 4H, BnS-CH_2_), 2.78–2.66 (m, 4H, HEA-CH_2_), 2.66–2.49 (m, 4H, HEA-CH_2_), 1.05 (s, 21H, TIPS-CH and
-CH_3_).

**((Diisopropylsilanediyl)bis(oxy))bis(ethane-2,1-diyl) diacrylate (DIPS-dA)**

**Scheme S5:** Synthesis of DIPS-dA.

2-Hydroxyethyl acrylate (500 µL, 553 mg, 4.76 mmol, 2.10 equiv.) and triethylamine (759 µL, 551 mg, 5.44 mmol, 2.40 equiv.) were dissolved in dry THF (4 mL) and the resulting mixture was cooled to 0 °C using an ice bath. Dichlorodiisopropylsilane (408 µL, 420 mg, 2.27 mmol, 1.00 equiv.) was dissolved in dry THF (4 mL) and added dropwise to the mixture at 0 °C for 30 min. Next, the reaction mixture was stirred at 0 °C for 30 min. Under subsequent stirring for 2 h, the mixture was allowed to warm to room temperature. Afterwards, the resulting suspension was filtrated and the residue was washed with THF (3 × 5 mL). The filtrate was washed with sat. aq. NaHCO_3_ (20 mL) and sat. aq. NaCl (20 mL), dried over Na_2_SO_4_, and dried *in vacuo* to afford DIPS-dA (775 µL, 775 mg, 2.25 mmol, 99%) as a colorless liquid.

**^1^H-NMR** (400 MHz, CDCl_3_, 295 K): δ = 6.42 (dd, *J* = 17.3, 1.5 Hz, 2H, HEA-CH=CH_2_), 6.13 (dd, *J* = 17.3, 10.4 Hz, 2H, HEA-CH=CH_2_), 5.83 (dd, *J* = 10.4, 1.5 Hz, 2H, HEA-CH=CH_2_), 4.30–4.23 (m, 4H, HEA-CH_2_), 4.02–3.95 (m, 4H, HEA-CH_2_), 1.06–1.00 (m, 14H, DIPS-CH and -CH_3_).

**^13^C-NMR** (101 MHz, CDCl_3_, 295 K): δ = 166.30 (C_q_, HEA-CO), 131.08 (CH_2_, HEA-CH=CH_2_), 128.43 (CH, HEA-CH=CH_2_), 65.73 (CH_2_, HEA-CH_2_), 61.12 (CH_2_, HEA-CH_2_), 17.24 (CH_3_, DIPS-CH_3_), 12.12 (CH, DIPS-CH).

*Experimental remarks and characterization procedures*

^1^H-NMR spectra of the synthesized P*o*PA polymers were recorded in DCM-d_2_. Due to the known acid sensitivity of polyacetals,^[2]^ the solvent was pretreated by washing with K_2_CO_3_, followed by filtration to remove trace acidic impurities and suppress unintended depolymerization during measurement. Under these conditions, the polymer backbone signals were clearly observable. However, the resonances corresponding to the terminal alcohol groups and the central silyl linker were significantly broadened and of comparatively low intensity, preventing reliable integration relative to the backbone and thus precluding accurate molecular weight determination. For the solution-state depolymerization experiment, THF-d_8_ was employed with equivalent pretreatment to ensure solvent compatibility with the TBAF trigger (added as a 1 m solution in THF).

To enable molecular weight determination, additional samples were prepared in CDCl_3_ without prior base treatment. In this solvent, trace acidic impurities induced complete depolymerization of the polymers within several hours. The resulting low-molecular-weight species exhibited sharp, well-resolved resonances, allowing reproducible integration of the relevant signals and calculation of the number-average molecular weight from the depolymerized samples. Independent measurements gave consistent results.

Furthermore, slight variations of Δ*DP*_n_ ≈ ±5 in degree of polymerization were obtained across different batches. For reasons of simplicity, all subsequent procedures and analyses are described for only three distinct chain lengths. The degree of polymerization *DP*_n_ differs among the polymers, with the following values: *DP*_n_ = 30, *DP*_n_ = 50, and *DP*_n_ = 116.

To prevent polymerization of the acrylated P*o*PAs on the column during SEC measurements, Michael additions with benzyl mercaptan (BnSH) were conducted beforehand. The procedure for the P*o*PA-dA variant utilized for 3D printing (*DP*_n_ = 50, *M*_n_ = 7.4 kDa) is described in the previous section as a representative protocol.

## S1.2. Solvent recovery, prepolymer architecture, and depolymerization

*Solvent recovery*

To maximize process sustainability of our established synthetic protocol for acrylated P*o*PAs,^[1]^ a modified work-up procedure was implemented to enable quantitative solvent recovery. After warming the reaction mixture to room temperature, THF was recovered via evaporation under reduced pressure (99% yield). Subsequently, the polymer was precipitated in EtOH, which was likewise recovered from the filtrate in near-quantitative yield (99%). The high purity of both recycled solvents was confirmed by ^1^H-NMR spectroscopy (Figure S1).

**Figure S1:** ^1^H-NMR spectra of recovered solvents. ^1^H-NMR spectra (301 MHz, CDCl_3_, 298 K) of THF and EtOH recovered from the synthesis and purification of P*o*PA-dA.

*Selective chain coupling and molecular weight control*

SEC was employed to confirm the coupling of two P*o*PA chains around the central silyl linker and the control over molecular weight by varying the initiator-to-monomer ratio (Figure S2).

**Figure S2:** SEC characterization and molecular weight control of P*o*PA-dA. **A** Comparative SEC traces of difunctional P*o*PA-dA (*DP*_n_ = 50) and monofunctional controls (mA) synthesized with matching (25:1) and double (50:1) initiator-to-monomer ratios. **B** SEC chromatograms of P*o*PA-dA variants with varying degrees of polymerization (target *DP*_n_ = 25, 50, and 100). Absolute molecular weights were determined by ^1^H-NMR spectroscopy.

Compared to monofunctional controls (P*o*PA-mA) synthesized according to our established protocols,^[1]^ the photoreactive P*o*PA-dA exhibited the expected doubling of molecular weight. Specifically, the difunctional species shows a significant shift toward lower retention volumes compared to a monofunctional control with an identical initiator-to-monomer ratio, while its elution profile nearly overlaps with a monofunctional control utilizing twice the monomer ratio (Figure S2A).

Furthermore, varying the initiator-to-monomer ratio enabled precise control over the molecular weight (*M*_n_ = 4.4–15.9 kDa) while maintaining remarkably narrow dispersities (*Đ*_M_ < 1.3), reflecting the high degree of living character of the anionic polymerization (Figure S2B). However, absolute molecular weights were determined via ^1^H-NMR spectroscopy, as SEC-derived values are influenced by differences in hydrodynamic volume and persistence length compared to the utilized PMMA calibration standards. Therefore, SEC was primarily employed to confirm the target bifunctional architecture and to verify the molecular weight control.

*Fluoride-triggered depolymerization in solution*

The responsiveness of the metastable P*o*PA-dA backbone to the defined chemical stimulus was validated via ^1^H-NMR spectroscopy using TBAF as a fluoride source (Figure S3).

**Figure S3:** ^1^H-NMR spectra of P*o*PA-dA before and after addition of TBAF. Addition of 2 equiv. of TBAF to a solution of P*o*PA-dA in THF-d_8_ leads to complete depolymerization within 2 min.

The addition of TBAF (2 equiv.) to a solution of P*o*PA-dA in THF-d_8_ at ambient temperature initiated the cleavage of the silyl trigger sites, resulting in a rapid, cascade-like depolymerization to the *o*PA monomer within less than two minutes. This quantitative transformation was confirmed by the complete disappearance of the broad resonance signals associated with the polymer backbone (7.75–6.32 ppm, corresponding to aromatic and acetal protons) and the simultaneous emergence of sharp signals characteristic of the *o*PA monomer (8.11–7.86 ppm and 7.84–7.55 ppm for aromatic protons, 10.49 ppm for aldehyde protons). This rapid transition from a stable macromolecule to its constituent building blocks confirms the high reactivity and kinetic efficiency afforded by the metastable, silyl-centered design (Figure S3).

# S2. Ink formulation and 3D printing performance

## S2.1. Ink formulations

**Ink 1**

First, P*o*PA-dA (1.24 g, 31.0 wt%, 1.1 mol%) was dissolved in DMI (2.19 mL, 2.52 g, 62.9 wt%, 87.9 mol%). Next, HEA (181 µL, 200 mg, 5.0 wt%, 10.5 mol%), BAPO (40 mg, 1.0 wt%, 0.6 mol%), and BHT (4 mg, 0.1 wt%, 0.1 mol%) were added. To ensure a complete solution, the ink was sonicated at rt for 30 min.

**Ink 2**

First, P*o*PA-dA (1.24 g, 31.0 wt%, 1.2 mol%) was dissolved in DMI (2.19 mL, 2.52 g, 62.9 wt%, 94.4 mol%). Next, DIPS-dA (200 µL, 200 mg, 5.0 wt%, 3.8 mol%), BAPO (40 mg, 1.0 wt%, 0.6 mol%), and BHT (4 mg, 0.1 wt%, 0.1 mol%) were added. To ensure a complete solution, the ink was sonicated at rt for 30 min.

**Ink 3**

First, P*o*PA-dA (1.24 g, 31.0 wt%, 1.1 mol%) was dissolved in DMI (2.19 mL, 2.52 g, 62.9 wt%, 90.9 mol%). Next, HEA (90.4 µL, 100 mg, 2.5 wt%, 5.4 mol%), DIPS-dA (100 µL, 100 mg, 2.5 wt%, 1.8 mol%), BAPO (40 mg, 1.0 wt%, 0.6 mol%), and BHT (4 mg, 0.1 wt%, 0.1 mol%) were added. To ensure a complete solution, the ink was sonicated at rt for 30 min.

**Note:** Among the synthesized library (*DP*_n_ = 30, 50, 116), the P*o*PA-dA variant with a degree of polymerization of 50 (*M*_n_ = 7.4 kDa) was found as the ideal candidate for ink formulation.

## S2.2. DLP 3D printability and lateral resolution assessment

*Evaluation of printing parameters*

To optimize the DLP process, the curing kinetics of inks 1–3 were quantitatively assessed using Jacob’s working curve model.^[3]^ By measuring the cure depth as a function of the exposure dose at a constant light intensity of 1 mW cm^–2^, we observed a systematic, crosslinking density-dependent decrease in the critical curing energy (*E*_c_ = 3.09–2.53 mJ cm^–2^) and an accompanying increase in the penetration depth (*D*_p_ = 119–168 µm) (Figure S4). These values underline the exceptional photosensitivity of the metastable ink platform, which enables energy-efficient processing even at low irradiation doses.

**Figure S4:** Jacob’s working curves for inks 1–3. The plots were obtained by evaluating the cure depth against the exposure energy at a constant light intensity of 1 mW cm^–2^.

*Lateral resolution assessment*

To evaluate the spatial fidelity of the metastable ink platform, a lateral resolution test was conducted using ink 1 as a representative system. The achievable resolution is determined by the intrinsic curing behavior of the ink and the hardware constraints of the digital micromirror device of the employed DLP printer. Given the printer’s pixel resolution of 27 µm, a test architecture featuring engraved line gratings with widths and periods of 108, 81, 54, and 27 µm (corresponding to four to one times the pixel size) was fabricated (Figure S5).

**Figure S5:** Lateral resolution test. Optical microscopy images (reflection mode) of engraved line gratings printed with ink 1. Feature sizes correspond to 108 µm (**A**), 81 µm (**B**), 54 µm (**C**), and the technical pixel limit of 27 µm (**D**). Scale bar: 50 µm.

Optical microscopy confirmed the exceptional structural fidelity of the ink, with all line widths and periods successfully resolved down to the technical limit of a single pixel (27 µm). Quantifying the line distances revealed a systematic reduction compared to the input STL dimensions, which is attributed to the isotropic shrinkage occurring during the development and drying process. Despite this effect, the precise reproduction of the grating periods validates the high spatial light control and minimal overcuring afforded by the metastable P*o*PA-based inks (Figure S5).

# S3. Comparison with recyclable 3D printable materials

To contextualize the performance of the metastable platform within the current state of the art, Table S1 compares our system with representative recyclable materials for light-based 3D printing.^[4–7]^ The comparison focuses on parameters that are consistently reported across these studies, namely tensile toughness, the nature of the degradable component and its breakdown mechanism, the applied trigger and conditions, and the chemical identity of the recovered species.

**Table S1**: Comparison of the metastable platform with representative recyclable materials for light-based 3D printing.

| **Author [Reference]** | **Toughness  [MJ m^–3^]** | **Degradable component (mechanism)** | **Breakdown trigger** | **Temperature [°C]** | **Time  [min]** | **Recovered species** |
| --- | --- | --- | --- | --- | --- | --- |
| **Sardon *et al.*^[4]^** | 0.72 | Thiourethane (derivatization) | Excess thiol, guanidine base | 25 | 180 | Oligomeric derivative |
| **Qi *et al.*^[5]^** | 1.73 | Poly-valerolactone (depolymerization) | Heat | 200 | 240 | Pure monomer |
| **Dove *et al.*^[6]^** | 4.50 | Poly-disulfide (depolymerization) | Phosphazene base, thiophenol | 80 | 180 | Monomer-oligomer mixture |
| **Xie *et al.*^[7]^** | 7.76–111.42 | Thioacetal  (partial dissociation) | Heat | 80 | 120 | Oligomers |
| ***This work*** | 1.93–2.60 | Poly-phthalaldehyde (depolymerization) | Fluoride | 25 | 1–5 | Pure monomer |

Within this group, the toughness of our printed networks is comparable to several established systems.^[4–6]^ While the thioacetal-based networks reported by Xie and coworkers reach higher toughness,^[7]^ these values are strongly formulation-dependent and are achieved through only partial network dissociation rather than complete monomer recovery.

The principal distinction of our platform lies in the combination of processing conditions and recovery fidelity. Depolymerization proceeds at room temperature within minutes, whereas the compared depolymerizable systems require elevated temperatures (80–200 °C) and substantially longer reaction times (2–4 h).^[5–7]^ Furthermore, several of these approaches rely on added catalysts or reagents,^[4,6,7]^ and afford oligomeric or oligomer-containing products,^[6,7]^ or a derivatized species rather than the pristine monomer.^[4]^ In contrast, the inherent metastability of the poly(*o*-phthalaldehyde) backbone enables complete, cascade-like depolymerization to the pure monomer under ambient conditions and without auxiliary reagents beyond the fluoride trigger.

# S4. Organogel development and mechanical profile

*Organogel vacuum and thermal stability*

To quantify the retention of DMI within the printed architectures, the mass evolution of a 3D printed strip (ink 1) was monitored during vacuum drying (< 1 mbar) and compared to a control previously developed in THF/EtOH to afford the solvent-free state. After 24 h of vacuum treatment, the mass stabilized at a plateau, indicating the formation of a stable organogel state. Comparison with the dry reference revealed a residual DMI content of approximately 50 wt%, which remains trapped within the network due to strong solvophilic interactions with the P*o*PA backbone (Figure S6A).

**Figure S6:** Vacuum and thermal stability of organogel. **A** Masses of 3D printed strips (ink 1) developed in DMI (organogel) or THF/EtOH (dry) upon vacuum drying. **B** TGA thermograms of materials (ink 1) developed in DMI (organogel) or THF/EtOH (dry).

The thermal stability of the organogel was further evaluated via TGA. In comparison to the solvent-free state, a characteristic decrease in the degradation onset temperature was observed (129 °C vs. 201 °C). This earlier weight loss upon heating is attributed to the evaporation of the entrapped DMI, while the subsequent degradation step corresponds to the thermal breakdown of the polymer matrix. These results underline that while DMI acts as an effective plasticizer, the underlying P*o*PA network retains its structural integrity until the solvent’s boiling point is approached (Figure S6B).

*Load-bearing tests*

To demonstrate the modulation of mechanical stiffness afforded by the development procedure, load-bearing tests were conducted on 3D printed buckyballs (ink 1). Vials filled with sand (5.1 g, exerting a force of ~50 mN) were placed on specimens in both the solvent-free and the organogel state. In the dry state, the buckyball supported the load without any perceptible deformation, consistent with the high storage moduli (> 600 MPa).

In contrast, the organogel state exhibited significant softening due to the high residual DMI content, which acts as a potent plasticizer within the P*o*PA network. This resulted in non-destructive, elastic compression of the architecture under the same load. These results visually confirm the ability to tailor the mechanical profile of the printed parts, ranging from rigid structural elements to soft, adaptive gel-like objects, simply by controlling the post-processing and drying routine (Figure S7).

**Figure S7:** Load-bearing tests of printed buckyballs. Photographs illustrating the mechanical response of architectures printed with ink 1 subjected to a static load (5.1 g sand-filled vial, ~50 mN). The solvent-free state maintains structural integrity without deformation, while the DMI-containing organogel state undergoes reversible compression.

# S5. Depolymerization trigger selectivity

To confirm the orthogonality and selectivity of the fluoride-triggered deconstruction, 3D printed buckyballs (ink 1) were immersed in various environments for 24 h. The architectures remained fully intact without detectable mass loss or degradation upon exposure to strong acidic (pH 2), strong basic (pH 12), or high ionic strength (5 m NaCl) aqueous solutions
(Figure S8A–C). Furthermore, immersion in pure THF resulted in pronounced network swelling without inducing deconstruction (Figure S8D), illustrating that solvophilic interactions and network expansion alone are insufficient to initiate the cascade. These results validate the high operational robustness of the metastable platform and confirm that depolymerization is exclusively coupled to the specific chemical cleavage of the silyl trigger sites.

**Figure S8:** Environmental stability tests. Photographs of 3D printed buckyballs (ink 1) after 24 h exposure to various conditions: **A** strong acidic (pH 2), **B** strong basic (pH 12), and **C** high ionic strength (5 m NaCl) aqueous solutions, as well as **D** pure THF.

# S6. Network breakdown mechanism and recovery efficiency

Upon exposure to the fluoride trigger, the printed networks disassemble into three distinct species, irrespective of the employed reactive modifier. Fluoride selectively cleaves the silyl ether bonds present both in the central lock of the P*o*PA-dA prepolymer and, where applicable, in the DIPS-dA crosslinker. This releases diisopropyldifluorosilane (DIPSF_2_) and initiates the cascade depolymerization of the metastable backbone to the pristine *o*PA monomer. The acrylate units, which form the covalent poly(acrylate) network during photopolymerization, are chemically inert toward the fluoride trigger and remain as a linear poly(2-hydroxyethyl acrylate) (poly-HEA) residue (Figure S9).

**Figure S9:** Schematic representation of the depolymerization and monomer recovery process.

Consequently, the three inks yield identical breakdown products, namely *o*PA, poly-HEA, and DIPSF_2_, and differ only in the relative quantities of the polyacrylate and the fluorinated silyl. During the subsequent purification (chromatography, filtration, or sublimation), these byproducts are separated from the *o*PA monomer, affording the pure crystalline monomer. The removal of the fluorinated silyl species was confirmed by ^19^F-NMR spectroscopy (Figure S32).

The theoretically recoverable *o*PA content of a printed object can be estimated from the ink composition. Since the solvent (DMI), photoinitiator (BAPO), and inhibitor (BHT) are removed during development, the mass of the cured object is defined by the covalently incorporated P*o*PA-dA and RM. Assuming mass-equivalent incorporation of all acrylated components, the recoverable *o*PA mass fraction of the printed object is given by:

$$\text{w}_{\text{o}\text{PA}}^{\text{theo}}\text{ =}\frac{\text{m}_{\text{P}\text{o}\text{PA}}^{\text{ink}}\text{×}\frac{\text{DP}_{\text{n,P}\text{o}\text{PA}}\text{×}\text{M}_{\text{o}\text{PA}}}{\text{M}_{\text{n,P}\text{o}\text{PA}}}\text{ }}{\text{m}_{\text{P}\text{o}\text{PA}}^{\text{ink}}\text{+}\text{m}_{\text{RM}}^{\text{ink}}}\text{ ×100\%.}$$

(1)

Since all three inks contain identical mass fractions of P*o*PA-dA (31.0 wt%) and RM (5.0 wt%), and share the same prepolymer, the theoretical *o*PA content is identical across all formulations and calculates to 82 wt%. This value is in close agreement with the experimentally recovered amounts of pure *o*PA relative to the total object mass (77 wt% for chromatography, 74 wt% for filtration, and 70 wt% for sublimation). These correspond to 85–94% of the theoretical maximum, with the minor deviations attributable to losses during the respective purification steps rather than incomplete depolymerization.

# S7. Reprinting and property retention of recycled prepolymers

To demonstrate that the recycled prepolymers retain their processing behavior and printing performance, the recycled P*o*PA-dA prepolymers (obtained through resynthesis after *o*PA recovery through all three isolation strategies) were combined and reformulated into an ink analogous to ink 1, which was then subjected to the same characterization workflow applied to the pristine material.

First, the curing kinetics of the reprinted ink were assessed using Jacob’s working curve model and compared directly to the pristine ink 1 (Figure S10). The recycled formulation exhibited a nearly identical curing behavior, with a critical curing energy of *E*_c_ = 3.23 mJ cm^–2^ and a penetration depth of *D*_p_ = 125 µm, closely matching the values obtained for the pristine ink (*E*_c_ = 3.09 mJ cm^–2^, *D*_p_ = 119 µm). This confirms that the photoreactivity and curing behavior of the ink are preserved upon recycling.

**Figure S10:** Jacob’s working curves of ink 1 formulated from pristine and recycled P*o*PA-dA, recorded at a constant light intensity of 1 mW cm^–2^. The nearly overlapping curves confirm the retention of the curing behavior upon recycling.

Second, the spatial fidelity of the reprinted ink was evaluated using the same lateral resolution test applied to the pristine material (Figure S5). Line gratings with widths and periods of 108, 81, 54, and 27 µm were fabricated and imaged by optical microscopy (Figure S11). All features were resolved down to the technical pixel limit of the DLP printer, and the measured line distances were nearly identical to those obtained for the pristine ink. This demonstrates that the high printing resolution is retained after recycling.

**Figure S11:** Lateral resolution test of the reprinted ink. Optical microscopy images (reflection mode) of engraved line gratings printed with ink 1 formulated from recycled P*o*PA-dA. Feature sizes correspond to 108 µm (A), 81 µm (B), 54 µm (C), and the technical pixel limit of 27 µm (D). Scale bar: 50 µm.

Finally, the triggered depolymerization of the reprinted material was compared to that of the pristine material. Buckyballs printed from pristine and recycled ink 1 were suspended in a TBAF/THF solution and their disassembly was monitored over time (Figure S12, Supplemental Movie 2). Both objects displayed an essentially identical deconstruction profile, progressing from the freshly suspended state through partial deconstruction to complete network dissociation within the same time frame. This confirms that the metastable, trigger-responsive character of the material is also preserved upon recycling.

**Figure S12:** Triggered depolymerization of pristine and reprinted buckyballs. Time-lapse photographs of buckyballs printed with ink 1 from pristine (A) and recycled (B) P*o*PA-dA, immersed in a TBAF/THF solution (1 m) and imaged after 0 s (freshly suspended), 150 s (partially deconstructed), and 300 s (fully deconstructed). Both objects exhibit an essentially identical deconstruction profile. The corresponding videos are shown side by side in Supplemental Movie 2.

Together, these results confirm that the recycled prepolymers reproduce the curing kinetics, printing resolution, and triggered depolymerization behavior of the pristine material across a complete recycling loop. In combination with the preserved molecular identity of the resynthesized prepolymers, evidenced by SEC (Figure S16–Figure S18) and 1H-NMR spectroscopy (Figure S33–Figure S38), these findings establish that the recycled materials are chemically identical to their pristine counterparts at every stage of the loop. This full recovery of the pristine building blocks is a direct consequence of the complete depolymerization enabled by the metastable backbone, which distinguishes the present approach from equilibrium-limited systems, where residual oligomeric content accumulates and progressively alters the composition and properties upon repeated recycling.

# S8. Appendix

**S8.1. SEC chromatograms**

**Figure S13:** SEC of P*o*PA-dBnS (*DP*_n_ = 30).

**Figure S14:** SEC of P*o*PA-dBnS (*DP*_n_ = 116).

**Figure S15:** SEC of P*o*PA-dBnS (*DP*_n_ = 50, synthesized from pristine *o*PA).

**Figure S16:** SEC of P*o*PA-dBnS (*DP*_n_ = 47, resynthesized after *o*PA purification through chromatography).

**Figure S17:** SEC of P*o*PA-dBnS (*DP*_n_ = 52, resynthesized after *o*PA purification through filtration).

**Figure S18:** SEC of P*o*PA-dBnS (*DP*_n_ = 49, resynthesized after *o*PA purification through sublimation).

**S8.2. NMR spectra**

**Figure S19:** ^1^H-NMR spectrum of P*o*PA-dA (*DP*_n_ = 30, 100 MHz, DCM-d_2_, 306 K); *H_2_O, ^#^grease.

**Figure S20:** ^1^H-NMR spectrum of P*o*PA-dA after depolymerization (*DP*_n_ = 30, 301 MHz, CDCl_3_, 299 K); ^+^acetone, *H_2_O, ^#^grease.

**Figure S21:** ^1^H-NMR spectrum of P*o*PA-dA (*DP*_n_ = 50, synthesized from pristine *o*PA, 100 MHz, DCM-d_2_, 306 K); *H_2_O, ^#^grease.

**Figure S22:** ^1^H-NMR spectrum of P*o*PA-dA after depolymerization (*DP*_n_ = 50, synthesized from pristine *o*PA, 301 MHz, CDCl_3_, 298 K); *H_2_O, ^#^grease.

**Figure S23:** ^1^H-NMR spectrum of P*o*PA-dA (*DP*_n_ = 116, 100 MHz, DCM-d_2_, 306 K); *H_2_O.

**Figure S24:** ^1^H-NMR spectrum of P*o*PA-dA after depolymerization (*DP*_n_ = 116, 301 MHz, CDCl_3_, 299 K); ^+^acetone, *H_2_O, ^#^grease.

**Figure S25:** ^1^H-NMR spectrum of P*o*PA-dBnS (*DP*_n_ = 50, 100 MHz, DCM-d_2_, 306 K); *H_2_O.

**Figure S26:** ^1^H-NMR spectrum of P*o*PA-dBnS after depolymerization (*DP*_n_ = 50, 301 MHz, CDCl_3_, 298 K); *H_2_O, ^#^grease.

**Figure S27:** ^1^H-NMR spectrum of DIPS-dA (400 MHz, CDCl_3_, 295 K); ^#^grease.

**Figure S28:** ^13^C-NMR spectrum of DIPS-dA (101 MHz, CDCl_3_, 295 K).

**Figure S29:** ^1^H-NMR spectrum of *o*PA (recovered through chromatography, 301 MHz, CDCl_3_, 298 K); *H_2_O, ^#^grease.

**Figure S30:** ^1^H-NMR spectrum of *o*PA (recovered through filtration, 301 MHz, CDCl_3_, 298 K); *H_2_O, ^#^grease.

**Figure S31:** ^1^H-NMR spectrum of *o*PA (recovered through sublimation, 301 MHz, CDCl_3_, 298 K); *H_2_O.

**Figure S32:** ^19^F-NMR spectra of *o*PA after plugging over silica, chromatography, filtration, and sublimation (183 MHz, CDCl_3_, 298 K); ^+^DIPSF_2_.

**Figure S33:** ^1^H-NMR spectrum of P*o*PA-dA (*DP*_n_ = 47, resynthesized after *o*PA purification through chromatography, 100 MHz, DCM-d_2_, 306 K); *H_2_O, ^#^grease.

**Figure S34:** ^1^H-NMR spectrum of P*o*PA-dA after depolymerization (*DP*_n_ = 47, resynthesized after *o*PA purification through chromatography, 301 MHz, CDCl_3_, 298 K); *H_2_O, ^#^grease.

**Figure S35:** ^1^H-NMR spectrum of P*o*PA-dA (*DP*_n_ = 52, resynthesized after *o*PA purification through filtration, 100 MHz, DCM-d_2_, 306 K); *H_2_O, ^#^grease.

**Figure S36:** ^1^H-NMR spectrum of P*o*PA-dA after depolymerization (*DP*_n_ = 52, resynthesized after *o*PA purification through filtration, 301 MHz, CDCl_3_, 298 K); *H_2_O, ^#^grease.

**Figure S37:** ^1^H-NMR spectrum of P*o*PA-dA (*DP*_n_ = 49, resynthesized after *o*PA purification through sublimation, 100 MHz, DCM-d_2_, 306 K); *H_2_O, ^#^grease.

**Figure S38:** ^1^H-NMR spectrum of P*o*PA-dA after depolymerization (*DP*_n_ = 49, resynthesized after *o*PA purification through sublimation, 301 MHz, CDCl_3_, 298 K); *H_2_O, ^#^grease.

**S8.3. Printing parameters**

**Figure S39:** Working curve of ink 1, formulated with P*o*PA-dA synthesized from pristine *o*PA.

**Figure S40:** Working curve of ink 1, formulated with P*o*PA-dA synthesized from recycled *o*PA.

**Figure S41:** Working curve of ink 2.

**Figure S42:** Working curve of ink 3.

**S8.4. TGA thermograms**

**Figure S43:** TGA thermogram of material printed with ink 1 in the dry development state.

**Figure S44:** TGA thermogram of material printed with ink 1 in the organogel development state.

**S8.5. DMA thermograms**

**Figure S45:** DMA thermogram of a strip printed with ink 1 in the dry development state.

**Figure S46:** DMA thermogram of a strip printed with ink 1 in the organogel development state.

**Figure S47:** DMA thermogram of a strip printed with ink 2 in the dry development state.

**Figure S48:** DMA thermogram of a strip printed with ink 2 in the organogel development state.

**Figure S49:** DMA thermogram of a strip printed with ink 3 in the dry development state.

**Figure S50:** DMA thermogram of a strip printed with ink 3 in the organogel development state.

**S8.6. Stress-strain diagrams**

**Figure S51:** Stress-strain diagram of a dumbbell-shaped specimen printed with ink 1 in the dry development state.

**Figure S52:** Stress-strain diagram of a dumbbell-shaped specimen printed with ink 2 in the dry development state.

**Figure S53:** Stress-strain diagram of a dumbbell-shaped specimen printed with ink 3 in the dry development state.

# S9. References

[1]	J. Markhart, P. Mainik, P. S. Klee, E. Blasco, "4D Printing of Self‐Immolative Polymers," *Adv. Funct. Mater.* 36, no. 23 (2026): e20642. https://doi.org/10.1002/adfm.202520642.

[2]	K. M. Lee, O. Phillips, A. Engler, P. A. Kohl, B. P. Rand, "Phototriggered Depolymerization of Flexible Poly(phthalaldehyde) Substrates by Integrated Organic Light-Emitting Diodes," *ACS Appl. Mater. Interfaces* 10, no. 33 (2018): 28062. https://doi.org/10.1021/acsami.8b08181.

[3]	P. F. Jacobs, *Fundamentals of Stereolithography*, Society of Manufacturing Engineers, Dearborn (1992):

[4]	X. Lopez de Pariza, O. Varela, S. O. Catt, T. E. Long, E. Blasco, H. Sardon, "Recyclable photoresins for light-mediated additive manufacturing towards Loop 3D printing," *Nat. Commun.* 14, no. 1 (2023): 5504. https://doi.org/10.1038/s41467-023-41267-w.

[5]	L. Yue, Y.-L. Su, M. Li, et al., "Chemical Circularity in 3D Printing with Biobased Δ-Valerolactone," *Adv. Mater.* 36, no. 34 (2024): e2310040. https://doi.org/10.1002/adma.202310040.

[6]	T. O. Machado, C. J. Stubbs, V. Chiaradia, et al., "A renewably sourced, circular photopolymer resin for additive manufacturing," *Nature* 629, no. 8014 (2024): 1069. https://doi.org/10.1038/s41586-024-07399-9.

[7]	B. Yang, T. Ni, J. Wu, et al., "Circular 3D printing of high-performance photopolymers through dissociative network design," *Science* 388, no. 6743 (2025): 170. https://doi.org/10.1126/science.ads3880.
